# Persistence With Biologic Therapy and Associated Costs of Patients With Inflammatory Bowel Disease: A German Retrospective Claims Data Analysis

**DOI:** 10.1093/crocol/otab011

**Published:** 2021-02-23

**Authors:** Joerg Mahlich, Melanie May, Chiara Feig, Vincent Straub, Renate Schmelz

**Affiliations:** 1 Health Economics and Outcomes Research, Janssen-Cilag GmbH, Neuss, Germany; 2 Düsseldorf Institute for Competition Economics (DICE), DICE, Heinrich-Heine-University Düsseldorf, Düsseldorf, Germany; 3 Health Economics, HGC Healthcare Consultants GmbH, Duesseldorf, Germany; 4 Medical Department, Universitätsklinikum Carl Gustav Carus, Technical University Dresden, Dresden, Germany

**Keywords:** inflammatory bowel disease, tumor necrosis factor α-inhibitors, treatment persistence, biologic therapy, real-world data, German claims data

## Abstract

**Background:**

In recent years, biologic agents became a relevant and promising treatment option for inflammatory bowel diseases (IBDs). However, high treatment costs and moderate remission rates lead to a high interest in treatment persistence and corresponding economic consequences.

**Methods:**

A retrospective health claims data analysis was conducted including biologic naive patients diagnosed with IBD between 2013 and 2018. Observation points were at 12 and 18 months of follow-up, starting from the first biologic prescription. Nonpersistence was defined as either no further prescription or prescription of another biologic agent within the days of supply per original prescription. Biologic agents included were Adalimumab, Golimumab, Infliximab, Ustekinumab, and Vedolizumab.

**Results:**

In total, 1444 patients with IBD were included in this analysis, mostly treated with Adalimumab (46.9%) and Infliximab (39.9%) as their first biologic treatment. After 12 months, 72.2% of patients were still persistent with their initial biologic treatment with the highest shares for Infliximab (74%) and Vedolizumab (72.4%). 27.8% of patients were nonpersistent, mostly due to a switch of biologic agent (75.8%). Cox regression identified female, hospitalizations, and simultaneous prescriptions of corticosteroids and immunomodulators as risk factors for nonpersistence. Treatment costs per year were approximately 3000€ higher for nonpersistent patients (27,146€) than for persistent patients (23,839€), mostly due to inpatient treatment costs.

**Conclusions:**

The persistence of biologic therapy in this study was rather high at 72% after 12 months, while nonpersistence was mostly due to switches to other biologic agents. Lack of persistence is associated with increased cost, mostly due to nonbiologic medication and inpatient treatment.

## INTRODUCTION

Inflammatory bowel diseases (IBDs) are characterized as intermittently recurrent or continuous inflammatory changes in the gastrointestinal tract, most commonly Crohn disease (CD) and ulcerative colitis (UC). The prevalence for IBD in Germany is estimated at over 300,000 cases per year and an incidence of 6.8 per 100,000 per year.^1,2^ The increasing prevalence and high social and economic burden makes it a growing global problem.^3^ Due to the predominantly early manifestation before the 30th year of life^4^ and the physically and socially challenging symptoms, such as chronic diarrhea, abdominal pain, anal bleeding, or severe internal cramps,^5,6^ patient’s quality of life is severely impacted.^7^ The exact causes for IBD are unknown, however current understanding suggests an exaggerated immune response to epithelial barrier dysfunction of the gut with bacteria penetrating the intestinal mucosa. Influencing factors are genetics,^8^ gut microbiota,^9^ and environmental disease-specific modifiers.^10^

According to the recent German AWMF S3-guidelines on therapy for UC and CD, mucosal healing and corticosteroid-free remission as well as endoscopic remission are relevant treatment targets,^5,6^ which leads to an increased quality of life for affected patients.^11^ However, these goals of clinical remission are achieved in about 30% of patients only.^12–16^ In recent years, targeted immunotherapy such as TNFα-inhibitors, anti-integrins, and IL12/IL23-inhibitors became an increasingly important therapy option for moderate and severe CD and UC.^17,18^ Approved for IBD therapy by the European Medicines Agency (EMA) are the following TNFα-inhibitors: Infliximab (originator since 1998, biosimilars since 2015), Adalimumab (originator: CD since 2003, CU since 2012, biosimilars since 2018), and Golimumab (indicated for UC only since 2013), the anti-integrin Vedolizumab (since 2014), the IL12/IL23-inhibitor Ustekinumab (CD since 2016, UC since 2019), and the JAK-inhibitor Tofacitinib (indicated for UC only since 2018). These therapy options can induce clinical remission, reduce hospitalizations, increase patient satisfaction, and might potentially prevent surgical intervention which often causes further complications.^19^ Due to the high social and economic burden caused by IBD and the fact that neither CD nor UC are curable,^5,6^ a continuously treatment is important for the long-term achievement of the defined therapy goals. This so-called treatment persistence is defined as the continuous prescription of 1 individual biologic agent over time. However, present studies indicated low persistence rates for patients with CD and UC under biologic treatment due to discontinuation or switch of therapy and also showed a great variety of databases and methods.^20–26^

Therefore, the aim of this study was to evaluate the treatment persistence of IBD patients under biologic treatment based on German real-world data (RWD) and the influential effect of potential risk factors. The ultimate goal was to evaluate the overall economic burden of IBD on the German health care system and the increased financial load due to nonpersistence.

## METHODS

### Data Source

This retrospective study was based on an anonymized routine dataset of ~5 million insurants from the German SHI claims data system, obtained from the research database of the Institute of Applied Health Research Berlin (InGef), which accounts for approximately 6% of the German population insured by SHI funds. The sample is representative of the German population in terms of age and sex and presents a good overall accordance of the database to the German population regarding measures of morbidity, mortality, hospitalization, and drug usage.^27^ Besides, the persistence of insurants within the database over time is high (78.5%), indicating suitability of the data source for longitudinal analyses.^27^ The database includes health care data from several SHI funds and covers 6 consecutive years from 2013 to 2018.

### Study Design and Patient Selection

This SHI claims data analysis was designed as a retrospective study with longitudinal cohort design and followed the eleven guidelines of the “Good Practice of Secondary Data Analysis” (GPS).^28^ Claims data were available from 2013 to 2018 and biologic naive patients with IBD were indexed from Q1 2014 to Q2 2017. The index date was defined as the first claim (prescription) of a defined biologic agent (Adalimumab, Golimumab, Infliximab, Ustekinumab, and Vedolizumab). Since the JAK-inhibitor Tofacitinib was first approved in August 2018 and the IL12/IL23-inhibitor Ustekinumab was not approved for UC until 2019, they are not considered in this study (years 2013–2018) for patients with UC. Both, prescriptions documented in the outpatient setting using the anatomical therapeutic chemical (ATC) codes and drug usage in the inpatient sector in form of the German operation and procedure (OPS) codes were considered. Biologic naive patients were identified as those who had no record of a biologic prescription 365 days (baseline 2013) before index date. Insurants with IBD were identified using the International Statistical Classification of Diseases, 10th Revision, German Modification (ICD-10-GM) of K50* (CD) and K51* (UC), as a confirmed outpatient or inpatient (main or secondary) diagnosis. Overlap patients with multiple recorded K50 and K51 ICD-10-GM diagnoses were assigned to either 1 group if 1 diagnosis (majority) was more frequently documented based on the last 9 diagnoses during index. Patients were excluded if no clear assignment was possible. Only continuously insured patients ≥18 years with confirmed CD or UC diagnosis were included to avoid loss to follow-up. Selected CD and UC patient groups were individually observed 12 and 18 months after index (Q3 2017–Q4 2018).

### Statistical Analysis and Outcomes

All outcome parameters were analyzed for the aggregated IBD group in comparison to the CD and UC study population and were compared on biologic level. Descriptive statistics were used to analyze patient characteristics and summarized in a patient flow. In addition, medical costs (EUR) divided into drug, outpatient, inpatient, medical aid, remedies, sick leave (sick leave days are defined as all days a person is not able to work based on doctor’s note. Costs for sick leave refer to all costs covered by health insurance. In Germany, during an employee’s illness, the agreed salary wage continues to be paid for a period of 42 days by the employer. After that health insurance covers sick pay for up to 546 days. Patients receive up to 90% of their net salary), and total costs were analyzed. Moreover, health care resource utilization (HCRU) was assessed for the outpatient (physician visits) and inpatient sector (hospitalization, length of stay), and sick leave (days/benefits). These outcome parameters were assessed preindex (12 months baseline) and postindex (12 and 18 months of follow-up). To ensure data privacy, quantities smaller than 5 were not displayed.

Treatment persistence for biologics after 12 and 18 months was observed, hereby patients were divided into either the persistence group, who continued index biologic treatment for the total observation period, or nonpersistence group due to (1) therapy switch, (2) therapy discontinuation, or (3) received only 1 prescription.

(1) patients switched from index biologic to another biologic within follow-up period,
(2) patients had prescription of index biologic but exceeded 60 days after the end of supply,
(3) patients showed only 1 prescription of index biologic.

Persistence status was calculated, both, for the in- and the outpatient sector. For the outpatient sector, the daily defined dosage (DDD in mg) for a male individual weighting 70 kg and 175 cm tall provided by the DIMDI, which refers to the official ATC/DDD definitions of the WHO,^29^ as well as the administered dose per prescribed German pharmaceutical registration number (PZN) were used to calculate days of supply, considering a therapy gap buffer of 60 days, and accordingly to assess persistence. Regarding the inpatient sector, the administered doses were either estimated on the lower boundary of the defined dose range per OPS coding or, if no dose ranges were available, the average maintenance dose for a male individual (70 kg and 175 cm) was calculated. These estimations were also multiplied with the associated DDD. Sensitivity analyses were performed with different definitions of persistence (30 vs 90 days vs last prescription) to examine how sensitive the results were with regard to the choice of the gap definition. To show persistence of different biologics, Kaplan–Meier curves were plotted on a weekly basis. A multivariate Cox regression was conducted to identify factors having an impact on nonpersistence (therapy discontinuation) of biologics, namely sex, age, Charlson comorbidity index (CCI) (mean number and differentiated into 3 groups: ≤2, 3–5, and >5), degree of polypharmacy (number of different ATC codes excluding predefined biologics; mean number and differentiated into 3 groups: ≤4, 5–9, and ≥10), insurance status (insured, pensioners, family-insured), and 3 dummy variables that indicate the presence of concomitant medications (steroids, immunomodulators), and hospital admissions (during follow-up). Moreover, separate Cox regressions were conducted testing the impact of the choice of biologic on persistence.

## RESULTS

### Study Population

**Figure 1.**
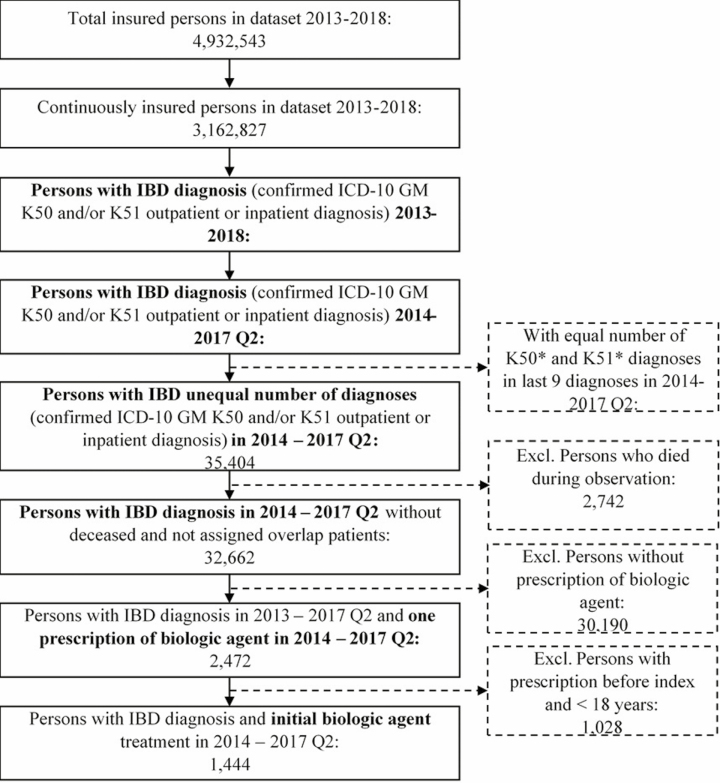
Study population flow chart.

Overall, 43,381 continuously insured prevalent patients with at least 1 confirmed outpatient or inpatient diagnosis of IBD (CD and/or UC) between 2013 and 2018 were identified in the database, which accounts for 722,000–1,126,000 patients extrapolated to the German population (6-year prevalence). A study cohort of 1444 therapy-naive patients with IBD diagnosis was identified who initiated biologic treatment between Q1 2014 and Q2 2017 (Fig. 1). Eight hundred forty-nine (58.8%) of those patients were assigned to the CD group and 595 (41.2%) to the UC group (including overlap patients).

### Patient Characteristics

In total, 677 patients initiated on Adalimumab (46.9%), 86 on Golimumab (6%), 576 on Infliximab (39.9%), 7 on Ustekinumab (0.5%), and 98 on Vedolizumab (6.8%). While 57.1% of CD patients were treated with Adalimumab (n = 485), for UC the share was at 32.3% (n = 192). Due to the assignment of overlap patients a small sample (<5) of patients with CD received Golimumab treatment, although it is not approved for this indication. The sex distribution was nearly equal between male (48.8%) and female (51.2%). Patients included were on average 42.7 years old. Comparing the different indications, the age distribution for UC patients was slightly higher with a mean age of 44.4, mostly due to a higher share of patients older than 50 years (36.1%) (Table 1).

**Table 1.** Patient Characteristics—Aggregated IBD Group in Comparison to the CD and UC Study Population

	IBD		CD		UC	
	n	%	n	%	n	%
Sex						
Male	704	48.8	394	46.4	310	52.1
Female	740	51.2	455	53.6	285	47.9
Age (mean ± SD)	42.71 (14.84)					
18–30 years	365	25.3	236	27.8	129	21.7
31–50 years	617	42.7	366	43.1	251	42.2
>50 years	462	32	247	29.1	215	36.1
Total	1444	100	849	100	595	100
CCI (mean ± SD)	0.46 (0.98)					
≤2	1383	95.8	817	96.2	566	95.1
3–5	53	3.7	29	3.4	24	4
>5	8	0.6	3	0.4	5	0.8
Total	1444	100	849	100	595	100
Polypharmacy (mean ± SD)	4.68 (2.91)					
≤4	629	55.4	405	59.6	224	49.2
5–9	422	37.2	232	34.1	190	41.8
>9	84	7.4	43	6.3	41	9
Total	1135	100	680	100	455	100
Insurance status						
Insured	1082	74.9	633	74.6	449	75.5
Pensioners	234	16.2	130	15.3	104	17.5
Family-insured	128	8.9	86	10.1	42	7.1
Hospitalizations (12 months)						
Yes	778	53.9	468	51.1	310	52.1
No	666	46.1	381	44.9	285	47.9
Prescriptions of steroids (12 months)						
Yes	925	64.1	500	58.9	425	71.4
No	519	35.9	349	41.1	170	28.6
Prescriptions of immunomodulators (12 months)						
Yes	767	53.1	331	39	436	73.3
No	677	46.9	518	61	159	26.7

The comorbidity of included patients, measured as the CCI, was relatively low with 95.8% of patients with 2 or fewer comorbidities. Patients received on average 4.7 pharmaceuticals (excluding biologics). Two of the included pharmaceuticals, steroids and immunomodulators, were specifically analyzed. 64.1% of patients had at least 1 prescription of a steroid and 53.1% of an immunomodulator during 12 months of follow-up. 53.9% of the included population were hospitalized at least once during the first 12 months of follow-up.

### Persistence

Overall, 72.2% (n = 1043) of IBD patients were persistent with their initial biologic treatment after 12 months and 62.9% (n = 908) after 18 months of follow-up. As a result, 27.8% of IBD patients were nonpersistent after 12 months and 37.1% after 18 months (Table 2). Among 401 nonpersistent patients, the main reason was a switch from initial biologic at 75.8% (n = 304) after 12 months followed by discontinuation at 16% and 1 prescription at 8.2%. Extrapolated to the total included population, approximately 2% of all patients discontinued after 1 prescription. For 18 months of follow-up, the share of patients discontinuing treatment increased to 19.4% (n = 104). Patients with UC showed a higher rate (86.1%) of nonpersistence due to a switch of biologic after 12 months, which led to a lower overall persistence rate after 12 months at 68.6% compared to CD at 74.8%. After 18 months, persistence rates for UC at 61.5% and for CD at 63.8% were balanced.

**Table 2.** Persistence Status and Reasons for Nonpersistence at 12 Months and at 18 Months

	IBD (n = 1444)	CD (n = 849)	UC (n = 595)
12 months			
Mean (±SD) (in days)	259.97 (102.22)	294.11 (102.95)	298.62 (101.20)
Not persistent (discontinuation/1 prescription/switch), n (%)	401 (64/33/304)	214 (46/25/143)	187 (18/8/161)
	(15.96%/8.23%/75.81%)	(21.5%/11.68%/66.82%)	(9.63%/4.28%/86.10%)
Persistent, n	1034	635	408
Persistent, %	72.2%	74.8%	68.8%
18 months			
Mean (±SD) (in days)	442.23 (163.26)	444.54 (161.58)	438.95 (165.72)
Not persistent (discontinuation/1 prescription/switch), n (%)	536 (104/31/401)	307 (77/23/194)	229 (27/8/194)
	(19.40%/5.78%/74.81%)	(25.08%/7.49%/67.43%)	(11.79%/3.49%/84.72%)
Persistent, n	908	542	366
Persistent, %	62.9%	63.8%	61.5%

Table 3 shows the persistence rate for 12 and 18 months of follow-up differentiated by biologic agent. After 12 months, persistence rates were at 74% for Infliximab, 72.4% for Vedolizumab, 72.12% for Adalimumab, and lowest for Golimumab at 66.3%. After 18 months, the shares for all biologics decreased. Vedolizumab had the second highest persistence rate after 18 months at 65.3% followed by Golimumab at 65.1%, Infliximab at 64.8%, and Adalimumab at 60.4%. Caution must be exercised for the findings regarding Ustekinumab. Even though the persistence rate was highest at 85.7% after 12 and 18 months, the small patient group treated with Ustekinumab (n = 7) is a limiting factor for comparisons of percentual shares. The main reason for nonpersistence on indication level was a switch of biologic; this was also found comparing different biologics, especially for Golimumab at 89.7% and Infliximab at 84% after 12 months. Adalimumab had the lowest rate of switches at 68%, but the highest rate of discontinuation at 24.2%, followed by Vedolizumab at 22.2% after 12 months. Two patients in the analysis were identified who initiated on Adalimumab and only had 1 prescription during 12 months of follow-up but had a prescription of another biologic between 12 and 18 months, which means they were assigned to the switch group after 18 months. This results in a lower number of nonpersistent patients with 1 prescription after 18 months compared to 12 months.

**Table 3.** Persistence Status and Reasons for Nonpersistence per Biologic Agent at 12 and 18 Months

	Total (n = 1444)	Adalimumab (n = 677)	Golimumab (n = 86)	Infliximab (n = 576)	Ustekinumab (n = 7)	Vedolizumab (n = 98)
12 months						
Mean (±SD) (in days)	259.97 (102.22)	295.49 (100.46)	280.73 (110.34)	294.25 (106.69)	267.57 (128.72)	324.78 (69.44)
Not persistent (discontinuation/1 prescription/switch), n	401 (64/33/304)	194 (47/15/132)	29 (0/<5/26)	150 (10/14/176)	<5 (<5/0/0)	27 (6/<5/20)
Persistent, n	1034	483	57	426	6	71
Persistent, %	72.2%	71.3%	66.3%	74%	85.7%	72.4%
18 months						
Mean (±SD) (in days)	442.23 (163.26)	448.05 (155.43)	412.99 (183.42)	434.64 (172.69)	384.57 (208.24)	476.46 (128.29)
Not persistent (discontinuation/1 prescription/switch), n	536 (104/31/401)	268 (79/13/176)	30 (<5/<5/27)	203 (13/14/176)	<5 (<5/0/0)	34 (11/<5/22)
Persistent, n	908	409	56	373	6	64
Persistent, %	62.9%	60.4%	65.1%	64.8%	85.7%	65.3%

**Figure 2.**
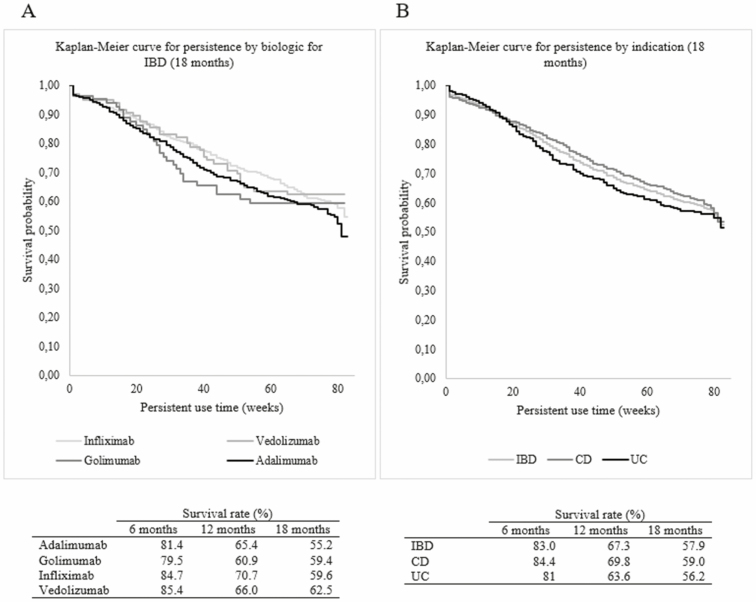
Kaplan–Meier plot for drug survival by biologics and IBD group (A) vs Kaplan–Meier plot for drug survival by UC, CD, and IBD group (B).

In addition to the persistence analysis, Kaplan–Meier curves were plotted, showing an overall drug survival for IBD patients of 57.9% after 18 months (Fig. 2). While the overall drug survival rate for CD was at 59% after 18 months, UC had a drug survival rate of 56.2%. This shows a 5% lower rate for both indications compared to the conducted persistence analysis. Comparing drug survival for the different biologic agents, Vedolizumab showed the highest rate at 62.5% followed by Infliximab at 59.6%, Golimumab at 59.4%, and Adalimumab at 55.2%. Ustekinumab was excluded from Kaplan–Meier analyses due to the relatively low population of 7 patients. The log rank *P*-value test conducted for the Kaplan–Meier curve comparing the different biologic agents showed no significant influence of the choice of biologic agent on the drug survival.

Findings from the Cox regression showed a significant influence of the patients’ sex on the persistence status; male patients seemed to have a lower risk for nonpersistence than women (Table 4). Moreover, hospitalizations and prescriptions of steroids or immunosuppressants were associated with nonpersistence. Findings indicated that patients with a prescription of steroid tend to have a 97% higher risk of being nonpersistent compared to patients without a prescription. For prescriptions of immunosuppressants, the risk was increased by 25.6% and for hospitalizations by 40.8%.

**Table 4.** Cox Regression Analyzing Risk Factors for Nonpersistence at 12 Months

	Parameter Estimate	SD	Chi-Square	Pr > ChiSq	Hazard Ratio	95% Hazard Ratio Confidence Interval	
Age							
18–30	−0.02679	0.14781	0.0333	0.8552	0.973	0.729	1.3
31–50	−0.10605	0.12931	0.6726	0.4121	0.899	0.698	1.159
>50	Reference						
Sex							
Male	−0.27334	0.1024	7.1253	0.0076	0.761	0.622	0.93
Female	Reference						
Insurance status							
Insured	0.24275	0.19369	1.5707	0.2101	1.275	0.872	1.863
Pensioners	0.42198	0.23024	3.359	0.0668	1.525	0.971	2.395
Family-insured	Reference						
CCI							
≤2	0.36466	0.2711	1.8093	0.1786	1.44	0.846	2.45
>2	Reference						
Hospitalization (yes)	0.34238	0.10305	11.0398	0.0009	1.408	1.151	1.723
Steroids (yes)	0.68026	0.11928	32.5266	<0.0001	1.974	1.563	2.494
Immunosuppressants (yes)	0.22795	0.10297	4.9003	0.0269	1.256	1.026	1.537

The conducted sensitivity analyses displayed that the overall persistence for IBD after 12 months was reduced to 62.5% by changing the defined therapy gap to 30 days, which in return increased the number of patients discontinuing treatment from 64 to 204. By considering a gap of 90 days, persistence slightly increased to 74.4%. When using a variable therapy gap definition based on the last prescription, only a minor change of persistence to 68.6% was found. These findings indicate a high robustness of the analyses’ results and the chosen persistence definition based on DDDs.

### Resource Utilization and Costs

On average total costs were at 24,760€ per patient after 12 months of follow-up (Table 5). Average biologic costs were estimated at 14,260€ and total drug costs at 17,900€ per patient. Total costs per patient were on average 3000€ higher for nonpersistent patients with 27,146€ compared to persistent patients with 23,839€. One impact factor for this is higher nonbiologic drug costs with 4347€ compared to 3378€ for persistent patients. Biologic drug costs were nearly identical for both groups with persistent patients at 14,237€ and nonpersistent at 14,333€, which leads to total drug costs of 18,680€ compared to persistent patients with 17,610€. The second highest cost item for both groups and biggest impact factor on the discrepancy between the persistent and nonpersistent patients was costs for inpatient treatments with 3620€ for persistent and 5089€ for nonpersistent patients. Altogether, the costs for nonpersistent patients were higher for all individual cost items compared to persistent patients.

**Table 5.** Costs (in EUR), HCRU, and Sick Leave at 12 Months

	Total		Persistent			Nonpersistent			*P* Persistent vs Nonpersistent	
	12 Months Pre	12 Months Post	12 Months Pre	12 Months Post	Δ Pre/Post	12 Months Pre	12 Months Post	Δ Pre/Post	12 Months Pre	12 Months Post
Mean costs in Euro € (±SD)										
Drug costs (biologics)	0.00€	14,263.77€	0.00€	14,237.01€	14,237.01€	0.00€	14,333.38€	14,333.38€	1	0.5837
	(±0.00)	(±8412.17)	(±0.00)	(±8480.95)		(±0.00)	(±8240.63)			
Drug costs (other)	1447.73€	3646.96€	1423.17€	3377.96€	1954.79€	1511.61€	4346.63€	2835.02€	0.1263	<0.0001
	(±2894.99)	(±7025.69)	(±2770.34)	(±6148.17)		(±3199.36)	(±8883.64)			
Drug costs (total)	1447.73€	17,907.64€	1423.17€	17,610.69€	16,187.52€	1511.61€	18,680.01€	17,168.40€	0.1263	0.0524
	(±2894.99)	(±8157.72)	(±2770.34)	(±6935.36)		(±3199.36)	(±10,674.58)			
Inpatient treatment	2568.41€	4027.92€	2491.44€	3619.84€	1128.40€	2768.61€	5089.33€	2320.72€	0.4819	0.0079
	(±5784.78)	(±9395.47)	(±5204.06)	(±7378.64)		(±7079.22)	(±13,231.03)			
Medical aids	75.59€	144.07€	76.98€	145.67€	68.69€	71.99€	139.91€	67.92€	0.304	0.0563
	(±331.84)	(±739.10)	(±352.92)	(±809.02)		(±269.74)	(±515.42)			
Outpatient treatment	1171.76€	1546.66€	1146.40€	1466.52€	320.12€	1237.71€	1755.10€	517.39€	0.1042	< 0.0001
	(±869.11)	(±967.09)	(±852.01)	(±921.74)		(±909.86)	(±1049.01)			
Remedies	64.74€	73.54€	67.13€	69.28€	2.15€	58.54€	84.61€	26.07€	0.6364	0.0739
	(±218.36)	(±231.43)	(±226.30)	(±229.86)		(±196.33)	(±235.39)			
Sick leave	715.56€	1057.51€	714.90€	926.97€	212.07€	717.27€	1397.06€	679.79€	0.63	0.08
	(±3768.98)	(±3957.99)	(±3986.88)	(±3722.00)		(±3136.60)	(±4501.80)			
Total costs	6043.79€	24,757.34€	5920.01€	23,838.97€	17,918.96€	6365.73€	27,146.03€	20,780.30€	0.4432	0.0001
	(±8669.20)	(±14,211.20)	(±8057.77)	(±10,349.48)		(±10,091.67)	(±21,014.72)			
Mean resource utilization (±SD)										
No. outpatient resource users (RU)	1439	1444	1039	1044		400	401			
No. outpatient visits per RU	28.07	34.95	27.19	33.36		30.09	39.08		0.0018	<0.0001
	(±15.66)	(±16.59)	(±15.64)	(±15.64)		(±16.67)	(±18.22)			
No. inpatient RU	918	778	663	540		255	238			
No. hospitalizations per RU	4.53	3.02	4.40	2.94		4.87	3.20		0.0907	0.0439
	(±3.85)	(±2.37)	(±3.74)	(±2.35)		(±4.11)	(±2.43)			
Length of stay per RU	19.74	26.33	19.09	25.73		21.41	27.68		0.8926	0.1598
	(±31.20)	(±47.46)	(±29.26)	(±49.09)		(±35.77)	(±43.61)			
Sick leave (mean ± SD)										
No. resource users (RU)	750	724	540	508		210	216			
No. sick leave days per RU	37.54	42.20	36.20	40.06		41.01	47.25		0.1822	0.0387
	(±37.71)	(±42.62)	(±36.04)	(±40.93)		(±41.60)	(±46.07)			

Analyzing the costs for biologic treatment on an individual level, findings show differences between the different agents, while Adalimumab (18,706€ for persistent compared to 16,372€ for nonpersistent), Golimumab (21,107€ for persistent compared to 16,732€ for nonpersistent), and Ustekinumab (22,469€ for persistent compared to 15,064€ for nonpersistent) all showed higher costs for persistent patients, Vedolizumab (9762€ for persistent compared to 14,922€ for nonpersistent) and Infliximab (8879€ for persistent compared to 11,121€ for nonpersistent) showed opposing results.

Analogous to the costs for inpatient treatment, the average number of hospitalizations was higher for nonpersistent patients with 3.2 hospitalizations compared to 2.94 for persistent patients with a longer average length of stay as well. Similar results were found for outpatient visits with 33.36 for persistent patients compared to 39.08 for nonpersistent and average number of sick leave days per patient with 47.25 for nonpersistent compared to 41.01 for persistent patients. Overall, after 12 months all patients had at least 1 outpatient contact. The number of patients with at least 1 hospitalization decreased from 918 to 778, as well as the number of patients utilizing sick days during 12 months of follow-up from 750 to 724.

## DISCUSSION

There is still limited evidence on the persistence to biologic treatment of patients with IBD in Germany. This retrospective longitudinal study provides information on persistence rates for 5 different biologic agents differentiated by indication (CD vs UC) using SHI claims data. Findings from this study reveal an overall high persistence to biologic treatment. Nonpersistent patients rather switch to another biologic agent than discontinue treatment. Moreover, nonpersistence is associated with increased overall costs, mostly due to inpatient treatment. Overall, findings indicate a higher burden of disease for patients not persistent to their initial biologic agent.

In comparison to other studies that analyzed biologic adherence for IBD patients, biologic persistence found in this study was rather high. A possible reason for this overall high persistence rate might be the inclusion of inpatient application of biologics using estimated ranges for OPS codes, since no known study analyzed biologic persistence for a German population with a similar approach. The increased number of prescriptions included in the analysis meant that the adherence could be examined more accurately as fewer patients were lost due to the choice of study design.

Another publication using German SHI claims data from 2019, for instance, found an overall persistence rate for IBD of approximately 62% after 12 months for biologic naive patients,^21^ which is nearly 10% lower than in this article. Another study by Chen et al reporting persistence rates for IBD patients based on RWD showed lower overall persistence as well. The study conducted in the United States found 1-year persistence rates for CD patients of 48.5% and 44.8% for UC, which is significantly lower compared to findings in this analysis (CD: 74.8%; UC: 68.6%). In addition to Chen et al including Certolizumab instead of Ustekinumab in their study, the findings for the other biologic agents analyzed in both studies showed some differences as well. While Chen et al showed the highest overall persistence for Adalimumab after 1 year, this study found Adalimumab to have the second lowest persistence rate. In the long run, however, patients with Infliximab analyzed in the Chen et al study presented the group with highest persistence. Golimumab showed the lowest persistence rate in both analyses.^20^ Differences in persistence rates could be due to the different measurement of persistence. While in this study the supply per prescription was based on DDDs, Chen et al used predefined days of supply per coding based on the American National Drug Codes (NDC) and Health Common Procedure Coding System (HCPCS). Since this calculation of days of supply is less flexible and only partly considers longer treatment periods per prescription, adherence could be underestimated.

Caution needs to be exercised analyzing the findings from this study since the defined washout period (baseline) of 1 year leaves the possibility that patients were already under biologic treatment before and treatment has been stopped because of sustained remission. This would include patients as biologic naive despite previous treatment. Due to the extended period between treatments, the response rate of these patients can be considered as biologic naive.^30^ Moreover, there was no investigation into other diagnosed diseases which could be treated with the included biologic agents, such as psoriasis, rheumatoid arthritis, or ankylosing spondylitis.

The sensitivity analyses performed, based on varied therapy gap definitions from 30 to 90 days as well as considering the maximum coverage of the last prescription, however, showed moderate to minor variation of the overall persistence, which indicates a high robustness of the study results.

An analysis of the reasons for nonpersistence suggests that findings are plausible. In general, according to current literature, only 50%–60% of IBD patients show a positive response to biologic treatment and the share of total remissions is only at approximately 30%.^12–16^ As a result, physicians change treatment patterns after 3–6 months at the latest if patients do not show improvement in relevant treatment targets. This could be an explanation for the high share of switches to other biologic agents in this study. The second reason for nonpersistence, discontinuation of treatment, is unlikely to be initiated by the patients themselves but mostly decided by the attending physician as a result of missing treatment effectiveness or severe side effects of biologic treatment. Another plausible reason for discontinuation could be a remission and a resulting de-escalation of biologic treatment. Since the patients included in this study are naive to previous biologic treatment, the switch to another biologic agent could be the more likely reaction to a failure of treatment and a complete discontinuation of biologic treatment is only seen in a few severe cases. Moreover, this naivety to biologic treatment could be an explanation for the fact that the Kaplan–Meier analyses conducted did not show a significant shift after 6 months.

Based on Cox regression, hospitalization (40.8% increased risk), prescriptions of steroids (97%), or immunomodulators (25.6%) were identified as higher risk factors for nonpersistence. Recent German AWMF S3-guidelines on therapy for UC and CD define higher quality of life without steroid prescriptions as a treatment target of biologic therapy.^5,6^ Thus, these findings on prescriptions and hospitalizations could indicate nonpersistence due to a failure of treatment rather than an actual negative impact of these factors on treatment adherence. Moreover, prescriptions of immunomodulators or corticosteroids are often used in combination with biologics, if the burden of disease for patients is overall very high, also implying a higher likelihood of treatment failure. The implication that male patients tend to be more persistent than women is consistent with a study published in 2020 by Greuter et al analyzing differences caused by sex for IBD.^31^ Findings from Greuter et al showed that female patients with IBD tend to be less adherent to treatment. A possible explanation is the more frequent occurrence of side effects for women than for men, resulting in a more frequent termination of treatment.

Insights into HCRU revealed increased total costs after 12 months compared to baseline. Compared to a study conducted by Wilke et al^32^ that analyzed treatment costs for IBD in Germany estimating 10,097€ for CD and 8772€ for UC annual total costs per patient, the total annual costs from this study (€24,757 per IBD patient) are high. Moreover, costs for inpatient treatment were at 4028€ per patient in this study, whereas Wilke et al found lower costs at 1072€ (CD) and 678€ (UC) per patient. A similar relation was found for the total annual drug costs. While Wilke et al estimated these costs at 4869€ for CD and 4621€ for UC, costs in this study were significantly higher at 17,908€. A possible explanation for this could be the different treatment patterns considered. While this study only included patients under biologic treatment, Wilke et al used different inclusion criteria regarding medications; biologic treatments were included but not required. For example, 42% of CD and 48% of UC patients initiated on nonbiologic immunosuppressant monotherapy. Since patients not under biologic treatment cause lower costs, treatment costs overall could be lower. Besides, this could be an indicator for a study population with a lower level of severity, due to the fact that biologics are mostly used as a second line therapy for moderate and severe patients during the last decade.

Moreover, the results showed that overall costs were significantly higher for nonpersistent patients compared to the persistent population, which corresponds to the findings from a Japanese study on rheumatoid arthritis.^33^ One main cost driver for this might be the high number of switchers in the nonpersistent group, assuming that inducing a new biologic therapy mostly requires higher initial doses and a higher frequency of admissions than maintaining therapy, which ultimately leads to higher costs.^34^ In addition, findings show an increased inpatient utilization for nonpersistent patients and higher resulting costs for inpatient treatment, accounting for the overall higher total costs for nonpersistent patients. Alongside the identification of hospitalizations as a risk factor for nonpersistence the increased inpatient costs indicate an overall higher morbidity for nonpersistent patients. These findings on hospitalizations are similar to the results of the recently published TRINordic study that analyzed real life data from Denmark, Norway, and Sweden between 2010 and 2016 regarding biologic use in IBD. The results published for hospitalizations also showed no general trend of biologics preventing inpatient utilization or surgical treatments.^35^ The trend of stable HCRU and sick leave days for resource users may be due to the therapy naivety of included patients since biologics are mainly used as second line treatment options. The previous failure of first line therapies and the resulting increasing burden of disease could be a cause for this development.^36,37^ Since the overall remission rate for patients with psoriasis is estimated at 90%–100%,^38^ for IBD remission is only seen for 30% of patients. As a result, the burden of disease is estimated to be higher and therefore the overall resource utilization and number of sick leave days might be higher for patients with severe symptoms.

The findings, that nonpersistence leads to increased medical costs, especially for inpatient treatment, are in line with a study from Wan et al^39^ examining the health care costs for IBD patients with Infliximab treatment. Wan et al identified an increase in inpatient costs associated with nonadherence to Infliximab treatment. Another study by Carter et al also analyzed the impact of nonadherence of patients with Infliximab treatment with CD,^40^ also identifying increased hospitalization costs for nonadherent patients compared to adherent. Caution needs to be exercised with regard to the fact, that persistence and adherence are not the same, but overall, these findings indicate an increased inpatient utilization and increased costs due to noncontinuous biologic treatment.

The second factor impacting the higher overall costs for nonpersistent patient is other (nonbiologic) drug costs. These costs can also be an indicator for a higher burden of disease for nonpersistent patients and a resulting necessity for more intensive additional therapy. Moreover, a failure of treatment resulting in a switch or discontinuation of treatment could lead to an additional need of therapy beyond biologic medication.

Generally, despite a generally large population, the sample sizes for the different biologic agents are vastly different. As a result, comparisons between the different agents need to be exercised with caution. Moreover, there was no further analysis conducted on why patients discontinued the biologic treatment. Since costs for biologic treatments are high, treatment de-escalation could be a possible measure to lower costs after disease remission and would be tracked as treatment discontinuation.

## CONCLUSIONS

In this study, the persistence of IBD patients to biologic therapy was rather high at 72% after 12 months, while switches to other biologic agents were the most common cause for nonpersistence. Since a switch of treatment is indicated after 3–4 months if no improvement in relevant treatment targets is seen and given that only 50%–60% of patients show a positive response to treatment and response rates can decrease over time, these findings seem to be plausible. Analysis of the total treatment costs showed overall higher costs for nonpersistent patients per year compared to nonpersistent, mainly due to nonbiologic drug costs and inpatient treatments. For a more detailed analysis of reasons for discontinuation and switch of treatment, further research is needed. Also, a more detailed analysis of impact factors for nonpersistence, especially psychiatric diagnoses, medication compliance, or other factors directly causing additional costs, shall be conducted.

## Data Availability

The datasets generated during and/or analyzed during the current study are not publicly available due to copyright issues but are available from the corresponding author on reasonable request.

## Notes

Funding: The study was funded by Janssen-Cilag, Neuss, Germany.

Conflict of Interest: J.M. was employed at Janssen-Cilag during conduct of the study and holds stocks from JNJ. M.M., C.F., and V.S. are employees of HGC, a company that received funding from Janssen-Cilag to conduct the study in collaboration with the Institute for Applied Health Research (InGef) in line with the study protocol. R.S. got no funding for this study, but received honoraria from Abbvie, Janssen, MSD, Takeda, and Pfizer for other lectures and consulting. All authors have no other relevant affiliations or financial involvement with any other organization or entity with a financial interest in the subject matter or materials discussed in the manuscript apart from those disclosed.

Ethical Approval: Not required as this study used anonymized German claims data.

Informed Consent: Not applicable.
